# Development and validation of the insulin treatment appraisal scale (ITAS) in patients with type 2 diabetes

**DOI:** 10.1186/1477-7525-5-69

**Published:** 2007-12-20

**Authors:** Frank J Snoek, Søren E Skovlund, Frans Pouwer

**Affiliations:** 1 Department of Medical Psychology, VU University Medical Center Amsterdam, The Netherlands; 2 EMGO Institute, VU University Medical Center Amsterdam, The Netherlands; 3 Novo Nordisk A/S, DAWN, Bagsvaerd, Denmark

## Abstract

**Background:**

Timely initiation of insulin therapy in type 2 diabetes is important to achieve metabolic control but can be hindered by negative perceptions of patients regarding insulin treatment. To assess the appraisal of insulin therapy of persons with type 2 diabetes, we developed the insulin treatment appraisal scale (ITAS) and tested its reliability and validity in insulin treated type 2 diabetes patients.

**Methods:**

A sample of 282 patients with type 2 diabetes form the United States (US) completed the ITAS, the WHO-5 Well-being index (WHO-5) and the Problem Areas in Diabetes (PAID) Survey. Exploratory factor analysis (EFA), internal consistency (Cronbach's alpha) and item-total correlations were determined to test the reliability of the instrument. Concurrent validity was examined by calculating Pearson correlation coefficients between the different measures. Discriminant validity was examined by comparing ITAS scores of insulin naive and insulin using patients.

**Results:**

EFA suggested a two-factor structure, separating positively worded and negatively worded items. Cronbach's alpha was 0.90 for the negative appraisal scale and 0.68 for the positive appraisal scale. Yet, Cronbach's alpha of the total 20-item scale was 0.89, suggesting high homogeneity and allowing for calculation of an overall score. Item-total correlations were in the range of 0.46–0.74 for the negative and 0.34 – 0.53 for the positive appraisal scale. The item pertaining to weight gain, as part of the negative appraisal subscale, showed low communality and deserves further testing. Concurrent validity was confirmed with low to moderate correlations in the expected direction between ITAS and WHO-5 and PAID. Discriminant validity was confirmed by the fact that patients using insulin had significantly less negative appraisals than insulin naive patients.

**Conclusion:**

The ITAS is a brief, psychometrically sound instrument that can be used in insulin naive and insulin-treated patients to assess positive and negative perceptions regarding insulin treatment and changes therein.

## Backgound

It is well recognized that intensive treatment can help to delay the onset of diabetes-related complications [1] and that many patients with type 2 diabetes require insulin therapy at some stage to achieve or maintain good glycaemic control [2]. In clinical practice however, initiation of insulin therapy is often delayed due to a variety of reasons, including patients' reluctance to accept insulin therapy [3,4]. The latter has been referred to in the literature as 'psychological insulin resistance' [5,6] a problem that was shown to be common among patients with type 2 diabetes in need of more intensive treatment [7,8]. Patients' reluctance to start insulin was found to be associated most strongly with the belief that starting insulin would indicate they had 'failed' to adequately self-manage their diabetes, next to fears about social stigma, perceiving insulin therapy as burdensome and too complex, worries about painful injections, the risk of hypoglycemia and anticipated weight gain [7-9]. To assist health care professionals and researchers in assessing barriers to timely insulin initiation and explore patients' attitudes towards insulin therapy, a short, comprehensive tool would be helpful. Moreover, such a measure would also be useful to prospectively measure changes in the patient's appraisals of insulin therapy in due course. For these purposes we developed the insulin treatment appraisal scale (ITAS), a 20-item self-report measure pertaining to both negative and positive beliefs regarding insulin treatment (see Additional file 1). Here we report on the development and validity of the ITAS.

## Methods

### Development of ITAS

The ITAS was developed to capture type 2 diabetes patient's current appraisal of insulin therapy. The instrument assesses both positive and negative attitudes. The respondent is asked to indicate on a 5-point Likert scale to what extent he or she agrees with each statement, from "strongly disagree to " strongly agree". Twenty potential items for the scale were generated from the literature on patients' barriers to staring insulin [8,9], discussions with diabetes care providers and clinical encounters with insulin naïve as well as insulin treated patients. The authors reached consensus on 4 positive and 16 negative statements (See Additional File 1). The ITAS was conceptualized as a two-dimensional instrument, with "appraisal of insulin therapy" as a single underlying construct, allowing for calculating a total score and two subscale scores. The ITAS has been designed as a diagnostic tool as well as an evaluation instrument to track changes in perceptions regarding insulin therapy over time.

### Patients

This validation study was conducted as part of a larger web-based survey on the impact of diabetes on treatment satisfaction, productivity and symptom experience conducted in the United States (US). The aims and methodology of this study were reported in detail by Brod et al. [10]. Briefly, participants were recruited from the Harris Interactive Chronic Illness Panel consisting of over 25,000 people with diabetes in the US who are considered a representative sample based on key characteristics for this population. From a total of 991 respondents who gave consent to receive the survey online, 282 type 2 diabetes patients participated in this sub-study (response-rate 29%), 146 insulin-naïve and 136 insulin-treated. The patient sample was obtained by use of quotas, i.e. recruitment was continued until there were equal number of insulin naïve and insulin-using patients. On average, the length of time since diabetes diagnosis of this sample was 5.1 years (SD 1.1, range 1–6).

### Measures

Socio-demographic and clinical data were self-reported as part of the online survey, using a short questionnaire. To ascertain the concurrent validity of the ITAS, patients were asked to fill in two validated and widely used psychological measures: the Problem Areas In Diabetes (PAID) scale and the World Health Organization Five Item Well-Being Index (WHO-5).

The PAID is a well validated and widely-used 20 items self-report scale to assess the current level of diabetes-related emotional distress both in type 1 and type 2 diabetes [11-13]. PAID items contain commonly expressed negative emotions related to living with diabetes (e.g. worrying about hypoglycemia, feeling burned out by the daily efforts to manage the diabetes, feeling worried about the future and complications) that are rated on a Likert scale ranging from 0 (not a problem) to 4 (a serious problem); scores are summed and standardized to a 0–100 scale, with higher scores indicating higher emotional distress.

The World Health Organization Five Item Well-Being index (WHO-5) is a uni-dimensional measure of emotional well-being containing five positively worded items [14]. The respondent is asked to indicate the degree to which these positive feelings were present in the last two weeks on a 6-point Likert scale, ranging from 0 (not present) to 5 (constantly present). Summation of the items scores provides a total score, which is standardized to a 0–100 scale. A higher score represents better emotional well-being. The WHO-5 has proven tot be a highly sensitive screener for depression in adults with and without diabetes [15,16].

### Statistical analysis

SPSS version 12.01 for Windows was used to analyse the data. Descriptive statistics were applied to calculate frequencies, means and standard deviation on all measures. Differences in demographic, clinical and questionnaire scores between the insulin naïve and insulin-treated group were tested using χ^2 ^tests to compare categorical data, and the Student's t-test or the Mann-Whitney U test for continuous data. Statistical significance was set at p < 0.05. Explorative factor analysis with Oblimin rotation was performed on the 20 ITAS items. An oblique rotation method was chosen because we anticipated that the factors would be correlated. The knick-criterion and the Kaiser-criterion (Eigenvalue > 1) were used to determine the optimal number of factors [17]. Next, the scree plot of the Eigenvalues was used to determine the optimal number of factors. Items with loadings exceeding 0.40 on one factor and less than 0.30 on any other factor are generally regarded as items with good scaling properties.

To assess the homogeneity of the retrieved scale(s), we calculated communalities, Cronbach's alpha, item-total correlations and inter-item correlations. For internal consistency, an alpha of 0.70–0.80 is desirable and the item-total correlation should be above 0.20. A high inter-item correlation (> 0.80) is often an indication of redundancy. Pearson correlations between total ITAS, PAID and WHO-5 scores were calculated as an indication for concurrent validity. It was hypothesized that PAID (emotional distress) would show a moderate positive association with negative appraisal of insulin therapy (*r *= 0.30–0.50). Lower WHO-5 scores (worse emotional well-being) were expected to be moderately associated with more negative appraisal of insulin therapy. Discriminant or known-groups validity was explored by comparing mean ITAS scores of insulin naïve versus insulin treated diabetes patients, expecting the latter group to report less negative appraisal, i.e. lower mean ITAS scores.

## Results

Complete questionnaires were available from 282 type 2 diabetes, of whom 136 (48%) were insulin treated. Self-reported characteristics of the male and female respondents are displayed in Table 1. Mean age in the total sample was 59 ± 11 years, 54% were female, mean HbA_1c _was 6.8 ± 1.8 and participants had a mean diabetes duration of 5 ± 1 years. Furthermore, the insulin naïve participants had a similar socio-demographic profile and ethnicity compared to subjects who were insulin-treated, but had a shorter disease duration (4.2 years versus 5.3 years, p < 0.001) and less often complications: cardiovascular disease (47% versus 57%, p < 0.05), eye/vision problems (21% versus 36%, p < 0.05) kidney problems (7% versus 11%, n.s.) and loss of feeling in hands or feet (21% versus 40%, P < 0.001). Not unexpectedly, Body Mass Index was significantly higher in patients who were treated with insulin, compared to the diet/tablet treated patients (33 vs. 36 kg/m^2^, p < 0.01).

**Table 1:** Self-reported demographic and clinical characteristics of the insulin naïve and insulin treated participants. * p < 0.05; ** p < 0.01; *** p < 0.001

	**Insulin naïve**	**Insulin-treated**
n (%)	146 (52%)	136 (48%)
Male sex	46% (67/146)	46% (63/136)
Living alone	31% (45/146)	24% (32/136)
White (Caucasian)	94% (135/144)	90% (120/133)
Age (years)		
30–49	19% (27/146)	27% (37/136)
50–64	38% (55/146)	34% (46/136)
65 or older	44% (64/146)	39% (53/136)
BMI	33 ± 7	36 ± 9 **
Highest education		
< high school	3% (4/146)	2% (2/136)
High school/GED	49% (72/146)	52% (70/136)
College degree	33% (48/146)	32% (43/136)
≥ Graduate degree	15% (22/146)	15% (21/136)
Treatment for type 2 diabetes		
oral medication	94% (137/146)	56% (76/136)***
insulin pump	-	2% (3/136)
HbA_1c_	6.5 ± 2.1 (n = 38)	6.9 ± 1.6 (n = 53)
Duration of diabetes (years)	4.2 ± 1.2	5.3 ± 0.8
Diabetes complications		
Retinopathy	21% (30/146)	36% (49/136)**
Cardiovascular	47% (68/146)	57% (77/136)
Nephropathy	7% (10/146)	11% (15/136)
Neuropathy	21% (31/146)	40% (55/136)***
Mean PAID total score	22 ± 22	30 ± 23 ***
Mean WHO-5	19 ± 5	18 ± 6**

### Factor analyses

Exploratory factor analyses (EFA) revealed 4 factors with an Eigenvalue >1. The first four Eigenvalues were 6.9, 2.2, 1.3 and 1.1. Using the knick-criterium, the drop in Eigenvalues after 6.9 and the "knick" in the plot after the second Eigenvalue suggests a uni-dimensional or 2-dimensional factor structure of the ITAS. In the unrotated 1 factor structure, the four positively worded items and the ITAS item on weigh gain had low communalities, ranging from 0.004 to 0.042. Using the Kaiser criterion, a maximum of 4 factors should be generated. Therefore, we calculated the 2-, 3- and 4-factor solutions for the ITAS using Oblimin oblique rotation (Table 2). The 2-factor structure consisted of 19 items: 15 negatively worded items loaded mainly on a first factor (F2.1) while the 4 positively worded loaded only on the second factor (F2.2). The "weight gain-item" had a very low communality (0.03) and did not load substantially on either of the two factors. This solution explained 45% of the total variance. Correlation between both factors was -0.04.

**Table 2:** Exploratory factor analyses of the 20 items of the ITAS: forced 2-, 3- and 4-factor solution after Oblimin rotation. Only factor loading > 0.40 are shown; *h*^2^: communality.

	2-factor solution:			3-factor solution				4-factor solution				
	
	*h*^2^	F1/2	F2/2	*h*^2^	F1/3	F2/3	F3/3	*h*^2^	F1/4	F2/4	F3/4	F4/4
**Item content:**												
1. Failed on pre-insulin therapy	0.34	0.59		0.53	0.45		0.67		0.71		0.82	
2. Diabetes has gotten worse	0.50	0.70		0.63	0.57		0.70		0.74	0.45	0.86	
3. Prevent complications	0.49		0.70	0.53		0.72		0.53		0.72		
4. Perceived by others as more sick	0.51	0.71		0.57	0.61		0.62		0.60	0.52	0.75	
5. Life less flexible	0.51	0.70		0.51	0.60		0.44			0.60		0.64
6. Fear of injecting with needle	0.40	0.62		0.52	0.70				0.52	0.70		
7. Risk of hypoglycaemia	0.28	0.52		0.28	0.48				0.39	0.50		0.44
8. Improves health	0.59		0.77	0.64		0.79		0.65		0.79		
9. Causes weight gain	0.03	-	-	0.33			0.60	0.80			0.88	
10. Takes time and energy	0.50	0.70		0.58	0.74				0.58	0.74	0.46	
11. Give up activities I enjoy	0.58	0.70		0.58	0.68				0.58	0.65	0.57	
12. My health will deteriorate	0.56	0.71		0.58	0.66		0.49		0.62	0.64	0.57	0.41
13. Injecting is embarrassing	0.53	0.70		0.57	0.73				0.57	0.72	0.49	
14. Injecting is painful	0.41	0.64		0.55	0.72				0.61	0.76		
15. Difficult to always inject correctly	0.49	0.70		0.58	0.76				0.59	0.77	0.41	
16. Difficult to fulfil responsibilities	0.67	0.80		0.71	0.82				0.71	0.81	0.56	
17. Helps to control blood glucose	0.55		0.74	0.56		0.73		0.56		0.72		
18. Family/friends more concerned	0.39	0.59		0.44	0.49		0.56		0.46	0.45	0.55	0.44
19. Helps to improve energy levels	0.28		0.53	0.28		0.53		0.29		0.53		
20. More dependent on doctor	0.47	0.67		0.48	0.62		0.47		0.49	0.56	0.63	

Explained variance:	45%			52%				57%				

In the 3-factor solution, all items had loadings > 0.40, yet 7 negatively worded items loaded on two factors. The 4-factor solution also included all 20 items, explaining 57% of the total variance. Like the 3-factor solution, this 4-factor solution showed many items loading on more than 1 scale. Ten items had double loadings and two items loaded on three factors.

The 2-factor solution would appear the best representation of the latent structure of the ITAS, given that all items had high communalities, except item 9 (insulin causes weight gain) and all positively worded items consistently loaded on one factor.

### Internal consistency

Cronbach's alpha was 0.89 for the 20-item scale, 0.90 for the 16-item negatively worded scale and 0.68 for the 4-item positively worded ITAS scale, indicating satisfactory homogeneity.

For the positive scale item-total correlations range from 0.34 to 0.53. In the negative scale, the item 'insulin causes weight gain' showed a low item-total correlation (0.10), while the remaining item-total correlations were in the range of 0.46 to 0.74.

### Concurrent validity

Pearson correlations between ITAS total (with positive scores reversed) and PAID (emotional distress) and WHO-5 (well-being) were 0.35 (p < 0.05) and -0.14 (p < 0.05) respectively, confirming low to moderate correlations in the expected direction. Higher ITAS scores (more negative appraisal) tend to go hand in hand with higher diabetes-related distress and lower emotional well-being. Additional analyses showed a comparable pattern of correlations with subscales: an association of 0.33 (p < 0.001) and -0.12 (p < 0.04) between ITAS-negative and PAID and WHO-5 respectively. For the positively worded ITAS subscale, correlations were -0.21 (P < 0.001) with PAID and 0.13 (p < 0.025) with the WHO-5.

### Discriminant validity

Mean scores and percentages of subjects who responded with 'agree' or 'strongly agree' to each of the 20 ITAS items are shown in Table 3, for insulin-naïve and insulin-treated patients. The mean total ITAS score of the insulin-naïve patients was about one standard deviation higher compared to insulin-treated patients (61.6 ± 12.8 vs. 48.9 ± 11.2, p < 0.001). Insulin-naïve patients reported significantly higher scores for all 16 negative items compared to insulin-treated, with the exception of the item pertaining to weight. Here 54% of the insulin-treated agreed that insulin causes weight gain, compared to 23% in the insulin naïve. The highest mean score for insulin-naïve patients was on the item pertaining to the belief that insulin signifies disease progression (item 2). Highest mean scores of the insulin-treated patients are on three of the four positive items (3, 8 and 17) pertaining to improved prognosis, improvement of health and good control of blood glucose, with 82%, 78% and 73% 'agree' to 'strongly agree' respectively. As to the item 'insulin helps to improve my energy levels' (item 19) both insulin-naïve and insulin-treated report relatively low agreement (25 and 35% respectively). The difference in responses to negative items between both groups is most striking on item 6 (fear of needle injections) where only 6% of the insulin-treated agrees to fearing injections compared to 47% of the insulin-naïve participants.

**Table 3:** Item content, mean scores and distribution of responses to individual items comparing insulin-naïve versus insulin treated participants and mean subscale and total ITAS scores. * p < 0.05; ** p < 0.01; *** p < 0.001.

	Insulin naïve (n = 146)		Insulin-treated (n = 136)	
	
	Mean (SD)	agree or strongly agree	Mean (SD)	agree or strongly agree
1. Insulin signifies failure with pre-insulin therapy	3.4 ± 1.4	54%	2.5 ± 1.4***	27%***
2. Insulin signifies diabetes has worsened	3.9 ± 1.6	73%	2.8 ± 1.3***	37%***
3. Insulin will improve prognosis	3.8 ± 1.1	62%	4.2 ± 1.0***	82%***
4. Insulin will make others perceive greater sickness	3.2 ± 1.3	41%	2.5 ± 1.2***	20%***
5. Insulin will make life less flexible	3.8 ± 1.1	70%	2.9 ± 1.3***	40%***
6. Fear of needle injection	3.1 ± 1.5	47%	1.4 ± 1.0***	6%***
7. Insulin will increase the risk of hypoglycaemia	3.1 ± 1.1	52%	3.0 ± 1.3***	40%***
8. Insulin will improve health	3.6 ± 1.0	53%	4.0 ± 1.0***	78%***
9. Insulin will cause weight gain	3.1 ± 0.9	23%	3.6 ± 1.3***	54%***
10. Insulin will be demanding to administer	3.6 ± 1.1	61%	2.7 ± 1.2***	28%***
11. Insulin means I have to give up activities I enjoy	2.6 ± 1.1	19%	1.9 ± 1.1***	10%***
12. Insulin means my health will deteriorate	2.7 ± 1.1	23%	2.2 ± 1.1***	13%***
13. Injecting insulin is embarrassing	2.6 ± 1.3	23%	1.8 ± 1.3***	10%***
14. Injecting insulin is painful.	3.3 ± 1.2	43%	2.7 ± 1.3***	38%**
15. It is difficult to always inject insulin correctly	3.2 ± 1.2	40%	2.3 ± 1.3***	26%***
16. Insulin makes it difficult to fulfil my responsibilities	2.9 ± 1.2	27%	1.9 ± 1.1***	9%***
17. Insulin helps to maintain good control of blood glucose	3.7 ± 1.0	59%	4.0 ± 1.1*	73%***
18. Using insulin causes family/friends to be more concerned	3.5 ± 1.1	55%	3.1 ± 1.3***	46%**
19. Insulin helps to improve my energy levels	3.2 ± 0.7	25%	3.1 ± 1.1 NS	35%***
20. Insulin makes me more dependent on my doctor	3.4 ± 1.1	40%	3.0 ± 1.2***	35%***

Mean Total Negative items ITAS	55.5 ± 12.7		44.1 ± 10.0***	
Mean Total Positive items ITAS	14.3 ± 2.9		15.2 ± 2.8*	

Mean Total ITAS (sum score 20 items, 4 negative recoded)	61.6 ± 12.8		48.9 ± 11.2***	

## Discussion

The findings from this study confirm good psychometric properties of the 20-item insulin treatment appraisal scale (ITAS) in both insulin naïve and insulin-treated type 2 diabetes patients. Factor analyses suggest a simple two-factor structure, with items pertaining to a positive and a negative appraisal of insulin. The internal consistency is high, suggesting the positive and the negative items relate to one underlying construct, namely a person's current appraisal of insulin therapy. Correlations between ITAS and PAID scale (diabetes-distress) were significant and in the expected direction, confirming concurrent validity. The linear association between ITAS and WHO-5 (well-being) was significant, but lower than expected. Interestingly, post-hoc analyses revealed that both among the insulin treated as well as the insulin-naïve patients, a WHO-5 score indicative of clinical depression (< 28) was associated with a significantly higher score on the ITAS (52.9 vs. 48.0, p = 0.046 and 65.5 vs. 60.9, p = 0.14 respectively). This finding is in concert with a recent study in Turkish insulin-naïve patients with type 2 diabetes where we found that symptoms of depression were positively associated with a more negative appraisal of insulin therapy [18]. Further research into the role of negative affect in patients' perceptions of insulin therapy is warranted, as depression is common among people with type 2 diabetes, adversely affecting self-management and subsequent clinical outcomes [19].

Data from this sample of US type 2 diabetes revealed some interesting differences between insulin-naïve and insulin-treated patients, suggesting a trend towards a less negative appraisal of insulin therapy in those who actually are on insulin treatment. The drop in fear of injections from 47% agreeing to 6% (mean score change from 3.1 to1.4) is most striking. Yet, still 36% of the insulin-treated patients agree to the item that injecting insulin is painful, compared to 43% in the insulin-naïve group. This suggests that despite improved injection devices and thinner needles, the daily injections are experienced as painful by a substantial number of patients. Prospective studies are warranted to determine the extent to which attitudinal changes towards insulin therapy occur in type 2 diabetes patients after insulin initiation.

Some limitations of this study need to be mentioned. First, the response rate in this cross-sectional study was rather low (29%) and may have influenced the results. Second, only persons with diabetes willing to complete the on-line surveys, with less than 10% of ethnic background, were included which may have further biased the outcomes. Future studies on the psychometric properties of the ITAS should include larger and more diverse samples of type 2 diabetes patients. This should also help to clarify the problem of low communality found for the item on weight gain in both insulin using and insulin naïve patients. The item apparently does not fit well in the scale and could be considered for deletion. However, Cronbach's alpha was not significantly improved when this particular item was removed, and the topic of weight gain is generally acknowledged as one of the important barriers in the current treatment of type 2 diabetes [20]. We therefore suggest to retain item 9 in the ITAS, deserving special attention. Interestingly, only 23% of the insulin-naïve patients agreed that insulin leads to weight gain, compared to 54% of the insulin-treated patients. Additional analyses showed that the answer "neither agree nor disagree" was the most common response (63%) in the insulin-naïve group, suggesting no clear view on the matter. In the insulin-treated group, however, only 27% responded neutral. Insulin-treated patients indeed had a significantly higher BMI, compared to insulin-naïve patients. Sixty % of the subjects with a BMI over 30 (obese) agreed that insulin use is associated with weight gain, compared to only 14–45% in the group of insulin users with a BMI lower than 30. These data suggest that weight gain as a result of insulin treatment is particularly an issue for those who already are overweight.

## Conclusion

The results from this study suggest that the ITAS is a valid self-report instrument that would seem useful in people with type 2 diabetes who have difficulty accepting insulin treatment. Examination and discussion of ITAS scores in clinical care can help to tailor education and treatment to the patient's needs. Also, the instrument has potential to assess changes in the appraisal of insulin over time, both in individuals and groups. Future research should establish its test-retest reliability and responsiveness.

## Abbreviations

BMI Body Mass Index

EFA Exploratory Factor Analysis

ITAS Insulin Treatment Appraisal Scale

PAID Problem Areas In Diabetes scale

WHO-5 World Health Organisation Five item Well-being Index

## Competing interests

This study was supported with an unrestricted grant from Novo Nordisk. FJS has received honoraria from Novo Nordisk for advisory services and non-commercial lectures. FP has received conference expenses from Novo Nordisk. SES is an employee of Novo Nordisk.

## Authors' contributions

SES participated in the conceptualisation and design of the original web-based survey. FJS, SES and FP assessed the quality of the data collected and participated in the design of the validation study and the development of the statistical plan. FP carried out the statistical analyses. All authors participated in the data interpretation. FJS drafted the manuscript to which SES and FP made significant contributions. All authors approved the manuscript submitted for publication.

## Supplementary Material

Additional file 1 Insulin Treatment Appraisal Scale (ITAS) Click here for file
