# Supplementary material for: Development and validation of the insulin treatment appraisal scale (ITAS) in patients with type 2 diabetes
Source: Health Qual Life Outcomes. 2007 Dec 20;5:69. doi: 10.1186/1477-7525-5-69 (PMC2241589; doi:10.1186/1477-7525-5-69)
Supplement: Additional file 1 — Insulin Treatment Appraisal Scale (ITAS) [file 1477-7525-5-69-S1.pdf]

Insulin Treatment Appraisal Scale (ITAS)

The following questions are about your perception of taking insulin for your diabetes. If you have not yet initiated insulin therapy, please answer each question from your current knowledge and thoughts about what insulin therapy would be like. Please indicate to what extent you agree or disagree with each of the following statements. Tick one box for each statement that best describes your own opinion.

	strongly disagree	disagree	agree nor disagree	agree	strongly agree
1. Taking insulin means I have failed to manage my diabetes with diet and tablets.	☐	☐	☐	☐	☐
2. Taking insulin means my diabetes has become much worse.	☐	☐	☐	☐	☐
3. Taking insulin helps to prevent complications of diabetes.	☐	☐	☐	☐	☐
4. Taking insulin means other people see me as a sicker person.	☐	☐	☐	☐	☐
5. Taking insulin makes life less flexible.	☐	☐	☐	☐	☐
6. I'm afraid of injecting myself with a needle.	☐	☐	☐	☐	☐
7. Taking insulin increases the risk of low blood glucose levels (hypoglycaemia).	☐	☐	☐	☐	☐
8. Taking insulin helps to improve my health.	☐	☐	☐	☐	☐
9. Insulin causes weight gain.	☐	☐	☐	☐	☐
10. Managing insulin injections takes a lot of time and energy.	☐	☐	☐	☐	☐
11. Taking insulin means I have to give up activities I enjoy.	☐	☐	☐	☐	☐
12. Taking insulin means my health will deteriorate.	☐	☐	☐	☐	☐
13. Injecting insulin is embarrassing.	☐	☐	☐	☐	☐
14. Injecting insulin is painful.	☐	☐	☐	☐	☐
15. It is difficult to inject the right amount of insulin correctly at the right time every day.	☐	☐	☐	☐	☐
16. Taking insulin makes it more difficult to fulfil my responsibilities (at work, at home).	☐	☐	☐	☐	☐
17. Taking insulin helps to maintain good control of blood glucose.	☐	☐	☐	☐	☐
18. Being on insulin causes family and friends to be more concerned about me.	☐	☐	☐	☐	☐
19. Taking insulin helps to improve my energy level.	☐	☐	☐	☐	☐
20. Taking insulin makes me more dependent on my doctor.	☐	☐	☐	☐	☐

Copyright Novo Nordisk and Frank Snoek
